# Response rates for providing a blood specimen for HIV testing in a population-based survey of young adults in Zimbabwe

**DOI:** 10.1186/1471-2458-7-145

**Published:** 2007-07-05

**Authors:** AD McNaghten, Joan M Herold, Hazel M Dube, Michael E St Louis

**Affiliations:** 1 Division of HIV/AIDS Prevention, National Center for HIV/AIDS, Viral Hepatitis, STD and TB Prevention, Centers for Disease Control and Prevention, Atlanta, USA; 2 Division of Reproductive Health, National Center for Chronic Disease Prevention and Health Promotion, Centers for Disease Control and Prevention, Atlanta, USA; 3 Department of Behavioral Sciences and Health Education, Emory University, Atlanta, USA; 4 Operations Research, Family Health International, Harare, Zimbabwe

## Abstract

**Background:**

To determine differences among persons who provided blood specimens for HIV testing compared with those who did not among those interviewed for the population-based Zimbabwe Young Adult Survey (YAS).

**Methods:**

Chi-square analysis of weighted data to compare demographic and behavioral data of persons interviewed who provided specimens for anonymous testing with those who did not. Prevalence estimation to determine the impact if persons not providing specimens had higher prevalence rates than those who did.

**Results:**

Comparing those who provided specimens with those who did not, there was no significant difference by age, residence, education, marital status, perceived risk, sexual experience or number of sex partners for women. A significant difference by sexual experience was found for men. Prevalence estimates did not change substantially when prevalence was assumed to be two times higher for persons not providing specimens.

**Conclusion:**

When comparing persons who provided specimens for HIV testing with those who did not, few significant differences were found. If those who did not provide specimens had prevalence rates twice that of those who did, overall prevalence would not be substantially affected. Refusal to provide blood specimens does not appear to have contributed to an underestimation of HIV prevalence.

## Background

Many countries in sub-Saharan African have conducted national population-based surveys that include HIV-1 testing. Although such surveys represent a major technical advance over convenience samples such as surveys in antenatal care settings, they introduce other technical issues. The representativeness of such surveys is an issue as exclusion of individuals who were not at home during the survey period or who refuse to participate can bias the results, possibly leading to an underestimation or overestimation of national HIV prevalence. The risk of HIV infection among persons who do not participate in the survey because they refuse or are not at home, or who agree to be interviewed but refuse to be tested may be different from those who consent to both the interview and HIV testing [1].

Among those who agreed to be interviewed in the Zimbabwe Young Adult Survey (YAS), we compared demographic and behavioral data from those who consented to and provided a blood specimen for anonymous HIV testing with those who declined to provide a specimen. We also estimated the impact on HIV prevalence if persons who did not provide a specimen had higher prevalence rates than those who provided specimens for testing to determine how this would impact overall prevalence rates.

## Methods

The Zimbabwe YAS was a nationally representative survey of men and women aged 15–29 years designed to estimate the prevalence of behaviors that may place young adults at risk for HIV, to evaluate the coverage and quality of services for HIV prevention and care, and to assess HIV prevalence among young adults in Zimbabwe. The design was a multi-stage household probability sample: the primary sampling units were 187 census enumeration areas in four geographic strata; the secondary sampling units were households in the selected census enumeration areas; and the tertiary sampling units were all eligible respondents in the selected households. In the second stage, households were randomly selected to belong to either the female sample or the male sample. Female interview teams went to households in the female sample and male interview teams went to households in the male sample to administer the household questionnaire. A roster in the household questionnaire identified eligible respondents living in the household; an eligible respondent in a female sample household would be a female aged 15–29 and in a male sample household a male aged 15–29. Household and individual interviews were conducted between September 2001 and February 2002. Filter paper was used to collect finger prick blood specimens, which were unlinked and tested anonymously for HIV antibodies. All persons interviewed were given a voucher for free HIV testing at a voluntary counseling and testing center and a transport subsidy. The Medical Research Council of Zimbabwe and the U.S. Centers for Disease Control and Prevention reviewed the protocol for data and specimen collection methods, including ethical considerations, and approved the YAS. Written informed consent was obtained from all participants who agreed to provide a blood specimen; additional parental consent was obtained for participants less than 16 years of age.

Interview data were weighted to adjust for the sample design and for non-response. The original sample was stratified by four geographic areas: the two major cities, Harare and Bulawayo, other urban areas, and rural areas. Because the sample design resulted in the oversampling of urban areas, weights were constructed to adjust for disproportionate stratification. Non-response weights were constructed using demographic data available from the household rosters collected during the household interviews. The sample design weights and the non-response weights were combined to produce a total weight for the individual respondents. With the exception of the response rates, all percentages and numbers presented were weighted by the total weights [2].

Chi-square analyses were performed using SAS version 8.2 (SAS Institute, Cary, NC) to compare demographic and behavioral data of persons interviewed who provided specimens for HIV testing with those who did not by age group, area of residence, education level, marital status, perceived risk of HIV infection, sexual experience and for sexually experienced respondents, number of lifetime sex partners.

To determine how prevalence estimates would be affected if persons who were interviewed but elected not to provide a specimen for testing had higher HIV infection rates, prevalence estimates were computed assuming persons who were interviewed but did not provide a specimen for testing had prevalence rates 2 times higher than those who provided a specimen. "Positives" were randomly assigned among those who did not provide a specimen.

## Results

**Figure 1:**
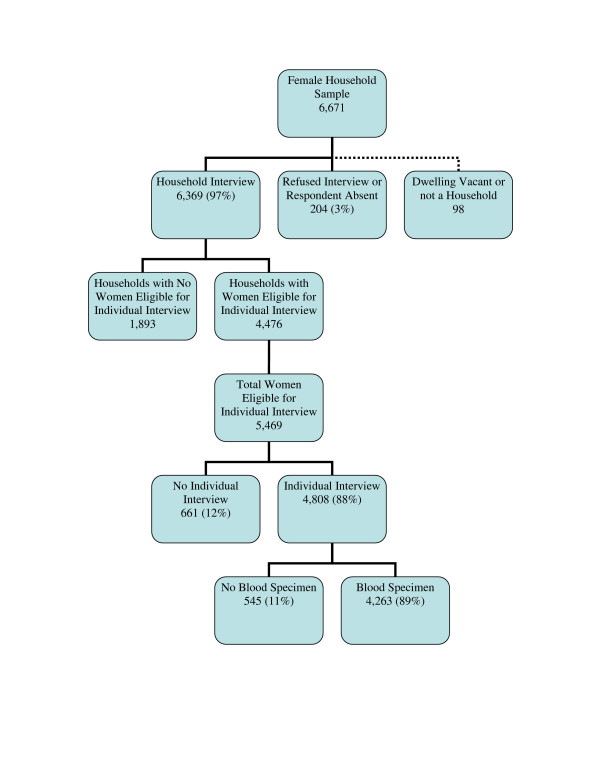
Household and individual response rates among females. Unweighted data.

**Figure 2:**
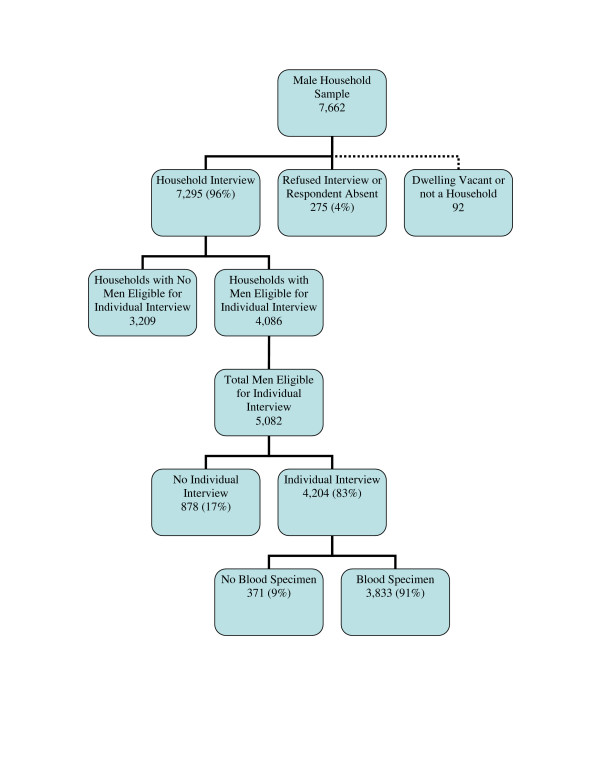
Household and individual response rates among males. Unweighted data.

The number of female and male household and individual questionnaires completed and acceptance or refusal of testing is outlined in Figures 1 and 2. Of 6,671 female and 7,662 male households visited, 97% of female and 96% of male households were interviewed. Among female households interviewed, 70% had at least one female eligible for individual interview and 30% did not have an eligible female. Among male households interviewed, 56% had at least one male eligible for individual interview and 44% did not have an eligible male. Of 5,469 eligible young women identified in the 4,476 female households, 88% were interviewed. Of 5,082 eligible young men identified in the 4,086 male households, 83% were interviewed. Eighty-nine percent of women and 91% of men interviewed also agreed to provide a specimen for HIV testing for an overall response rate (household interview rate multiplied by individual interview rate multiplied by blood specimen rate) of 76% among women and 72% among men.

Of female households selected, 1.6% had residents who were not at home, 1.5% was vacant, destroyed or not found and 1.0% refused. Of male households selected, 1.3% had residents who were not at home, 1.2% was vacant, destroyed or not found and 1.6% refused.

Among women who were selected for individual interview, the nonresponse comprised 4.6% refusals, 4.4% not at home, 1.2% incapacitated and 2.0% incomplete interviews. Among men selected for individual interview, nonresponse comprised 4.5% refusals, 9.7% not at home, 1.1% incapacitated and 2.3% incomplete interviews.

When comparing women who provided a specimen for HIV testing and those who did not, we found no significant difference by age group, residence, education level, marital status, perceived risk of HIV infection, sexual experience or number of lifetime sex partners (Table 1). Comparing men who provided a specimen for HIV testing with men who did not, we found a significant difference by sexual experience (Table 2). A larger percentage of men who provided a specimen for testing reported they had ever had sex. There was no significant difference between men who provided a specimen for testing and men who did not by age group, area of residence, education level, marital status, perceived risk of HIV infection or number of lifetime sex partners.

**Table 1:** Provision of blood specimen for anonymous HIV testing among women who were interviewed.

	% Provided Specimen (No.) weighted n = 4293	% Did Not Provide Specimen (No.) weighted n = 517	p-value
**WOMEN**			
**Age Group**			0.4137
15–19	42.1 (1807)	39.3 (203)	
20–24	32.5 (1395)	35.0 (181)	
25–29	25.4 (1091)	25.7 (133)	
**Area of Residence**			0.0733
Urban	38.0 (1629)	42.0 (217)	
Rural	62.0 (2664)	58.0 (300)	
**Education Level**			0.0835
Primary or less	33.3 (1428)	34.2 (177)	
1–3 years secondary	32.0 (1374)	27.4 (142)	
≥ 4 years secondary	34.7 (1491)	38.4 (199)	
**Marital Status**			0.8701
Previously Married	8.2 (352)	8.9 (46)	
Currently Married	46.2 (1985)	45.8 (237)	
Never Married	45.6 (1956)	45.3 (235)	
**Perceived Risk of HIV Infection**			0.0574
None	56.8 (2288)	62.6 (303)	
Low	12.6 (507)	13.0 (63)	
Medium	13.4 (539)	10.4 (50)	
High	7.6 (304)	7.2 (35)	
Don't Know	9.5 (382)	6.8 (33)	
**Sexual Experience**			0.4795
Ever had sex	66.1 (2834)	64.6 (334)	
Never had sex	33.9 (1455)	35.4 (183)	
**Number of Lifetime Sex Partners**			0.8990
1	71.0 (2014)	71.8 (239)	
2	19.8 (563)	18.8 (62)	
3 or more	9.2 (260)	9.4 (31)	

**Table 2:** Provision of blood specimen for anonymous HIV testing among men who were interviewed.

	% Provided Specimen (No.) weighted n = 3844	% Did Not Provide Specimen (No.) weighted n = 356	p-value
**MEN**			
**Age Group**			0.2277
15–19	43.0 (1653)	47.8 (170)	
20–24	29.3 (1125)	27.0 (96)	
25–29	27.7 (1065)	25.3 (90)	
**Area of Residence**			0.2759
Urban	41.4 (1591)	44.4 (158)	
Rural	58.6 (2253)	55.6 (198)	
**Education Level**			0.0830
Primary or less	21.9 (842)	27.1 (96)	
1–3 years secondary	30.3 (1164)	28.6 (102)	
≥ 4 years secondary	47.8 (1838)	44.3 (158)	
**Marital Status**			0.1210
Previously Married	2.2 (86)	0.6 (2)	
Currently Married	21.1 (812)	21.3 (76)	
Never Married	76.7 (2946)	78.1 (278)	
**Perceived Risk of HIV Infection**			0.1849
None	73.2 (2729)	72.3 (243)	
Low	11.8 (439)	12.4 (42)	
Medium	8.4 (312)	7.0 (24)	
High	4.8 (178)	4.4 (15)	
Don't Know	1.9 (72)	3.8 (13)	
**Sexual Experience**			0.0026*
Ever had sex	62.5 (2401)	54.4 (194)	
Never had sex	37.5 (1443)	45.6 (163)	
**Number of Lifetime Sex Partners**			0.1655
1	30.6 (733)	36.7 (71)	
2	20.8 (498)	17.1 (33)	
3 or more	48.6 (1165)	46.2 (90)	

*p < 0.05

Overall estimates of HIV prevalence assuming those who were interviewed but did not provide a specimen for HIV testing had a prevalence rate 2 times higher than those who did provide a specimen for testing were not substantially higher than the overall prevalence based on women and men aged 15–29 years who provided specimens (Table 3). Estimates of prevalence would increase from 21.8% to 24.2% among women and from 10.3% to 11.1% among men. This effect is also shown by age group for women and men in Table 3. It is important to note that if respondents who did not provide a specimen had a *lower *prevalence, say, 2 times lower than those who did provide a specimen, the decrease in total prevalence would produce the same magnitude of difference as the increase but in the opposite direction (data not shown).

**Table 3:** Effect of higher prevalence among interviewees who did not provide a blood specimen for HIV testing on YAS prevalence estimates by sex.

	Prevalence (%)	If persons not providing a specimen were 2x higher (%)
**WOMEN**		
**Age Group**		
15–19	10.6	11.7
20–24	26.1	29.1
25–29	34.7	38.5
**Total (15–29)**	21.8	24.2
		
**MEN**		
**Age Group**		
15–19	2.1	2.3
20–24	9.2	9.9
25–29	24.4	26.3
**Total (15–29)**	10.3	11.1

## Discussion

Both female and male household interview response rates were high (97% and 96%, respectively) compared with other national population-based surveys in sub-Saharan Africa (75.4%–99.7%) [3][4]. Individual interview response rates were relatively high as well. Similar to other population-based surveys conducted in African countries during this same time period, overall response rates declined when HIV testing was included [3].

Gregson et al. found that HIV prevalence was higher in working men in rural Zimbabwe [5], and it is possible that some of the men who were not interviewed may have been working away from home. Lydie et al. found that among men in Cameroon, as time away from town increased, so did HIV prevalence [6]. However, individuals who were not interviewed because they were not at home during the survey period or refused to participate should not have biased the sample because these data were weighted to adjust for such non-response. This weighting to adjust for non-response should have ensured the representativeness of the survey.

We examined these data to determine if there were any potential sources of bias that could have resulted in the underestimation or overestimation of HIV prevalence in the YAS. We found no significant demographic or behavioral differences among women who provided a specimen for testing and women who did not. The only significant finding among men was by sexual experience. Among men who provided a specimen, a larger percentage reported ever having sex. In the YAS, HIV prevalence among sexually experienced men was over 5 times higher than men who reported never having sex [2]. The overrepresentation of sexually experienced males in the group that provided specimens therefore could have contributed to an overestimation of HIV prevalence.

If those interviewed but not providing a specimen for HIV testing had prevalence rates 2 times higher or 2 times lower than those who provided specimens, YAS prevalence estimates would not be greatly affected. The magnitude of difference in prevalence for those providing specimens versus those electing not to was greatest in the 25–29 year age group for both women and men.

High rates of infection among women aged 15–29 years attending antenatal clinics (ANC) (24.6%) have been documented by Zimbabwe's ANC sentinel surveillance program [7]. However, there are limitations of ANC sentinel surveillance, including a lack of HIV prevalence data on men, non-pregnant women, women receiving care from private providers and non-sexually active women. Studies in sub-Saharan Africa have shown that HIV prevalence in pregnant women frequently underestimates prevalence in women of reproductive age in the general population [8], are generally higher than the prevalence in men [9] but fairly accurately represents prevalence of both men and women in the general population [10]. Periodic population-based surveys can serve not only to provide reasonably accurate estimates of HIV prevalence to supplement sentinel surveillance data, but can help validate estimates obtained from sentinel sites or produced using mathematical models. Similarities in the Zimbabwe ANC and YAS prevalence data are probably due to the high ANC attendance in the country [11]. Of the women in the YAS who reported being pregnant in the five years prior to being interviewed, 95.0% reported receiving antenatal care [2].

Although there are many advantages of using a population-based survey to estimate HIV prevalence among young Zimbabwean adults, there were some limitations in the YAS and this analysis. Reasons for not providing a specimen for HIV testing were not collected, but likely reasons include fear of the procedure and fear that confidentiality may not be upheld. Because the sample was drawn from households, police, military and incarcerated populations were not included in the YAS. The prevalence of HIV in young adults in these populations may be very different than that of the general population.

When comparing demographic and behavioral characteristics of persons who provided specimens for HIV testing with those who did not, few significant differences were found that could have potentially contributed to overestimating or underestimating HIV prevalence in our study population. If those who did not provide specimens had prevalence rates even twice (or one-half) that of those who did, prevalence would not be substantially affected. Therefore, the estimates obtained from the YAS appear representative of the population aged 15–29 years in Zimbabwe.

## Competing interests

The author(s) declare that they have no competing interests.

## Authors' contributions

AM conceived the study, its design, and drafted the manuscript. JH performed analyses and contributed to the writing of the manuscript. HD and MS contributed to the content and writing. All authors read and approved the final manuscript.

## Pre-publication history

The pre-publication history for this paper can be accessed here:


